# Comparison of Gene Expression Profiling in Sarcomas and Mesenchymal Stem Cells Identifies Tumorigenic Pathways in Chemically Induced Rat Sarcoma Model

**DOI:** 10.5402/2012/909453

**Published:** 2012-07-17

**Authors:** Kanya Honoki, Hiromasa Fujii, Yasuaki Tohma, Toshifumi Tsujiuchi, Akira Kido, Shinji Tsukamoto, Toshio Mori, Yasuhito Tanaka

**Affiliations:** ^1^Department of Orthopedic Surgery, Nara Medical University, 840 Shijo-cho, Kashihara 634-8521, Japan; ^2^Department of Life Science, Faculty of Science and Technology, Kinki University, Higashiosaka 577-8502, Japan; ^3^RI Center, Nara Medical University, Kashihara 634-8521, Japan

## Abstract

Mesenchymal stem cells (MSCs) are believed to be the cell of origin for most sarcomas including osteosarcoma and malignant fibrous histiocytoma (MFH/UPS). To identify the signaling pathways involved in sarcoma pathogenesis, we compared gene expression profiles in rat osteosarcoma and MFH cells with those in syngeneic rat MSCs. Analysis of genes that characterize MSCs such as CD44, CD105, CD73, and CD90 showed higher expression in MSCs compared to sarcomas. Pathways involved in focal and cell adhesion, cytokine-cytokine receptors, extracellular matrix receptors, chemokines, and Wnt signaling were down-regulated in both sarcomas. Meanwhile, DNA replication, cell cycle, mismatch repair, Hedgehog signaling, and metabolic pathways were upregulated in both sarcomas. Downregulation of *p*21^*Cip*1^ and higher expression of CDK4-cyclinD1 and CDK2-cyclinE could accelerate cell cycle in sarcomas. The current study indicated that these rat sarcomas could be a good model for their human counterparts and will provide the further insights into the molecular pathways and mechanisms involved in sarcoma pathogenesis.

## 1. Introduction

 Sarcomas are considered to be malignant neoplasms of mesenchymal origin, occurring mainly in bone and soft tissues such as muscles and adipose tissue. There are more than 100 recognized histopathological subtypes [1], yet they represent only approximately 1.5–2% of malignancies diagnosed annually. Among them, osteosarcoma is the most common malignant bone tumor, with a yearly incidence of approximately 6 per million children and 2 per million adults [2]. The outcome for patients with high-grade osteosarcoma has improved substantially since the introduction of multimodal chemotherapy, with present overall survival rates ranging from 65 to 75%.

Malignant fibrous histiocytoma (MFH) has previously been one of the most frequently diagnosed sarcoma types. However, this diagnostic category has been reorganized and is now described as undifferentiated pleomorphic sarcoma (UPS), representing a relatively small entity in soft tissue sarcomas [3]. Although UPS may not represent a distinct biological entity, it may share patterns of gene expression with heterogeneous high-grade karyotypically complex sarcomas [4]. 

The multidisciplinary strategies used to combat sarcomas consisting of surgery combined with chemotherapy and radiotherapy have resulted in a substantial improvement in patients' outcomes. However, the improvement seems to reach a plateau, and 30–40% of the patients still die of the disease. Since limited effective therapeutic options are available for those patients with poor prognosis, the need to understand the pathogenesis as well as molecular biological characteristics has emerged to facilitate the development of new diagnostic markers and therapeutic agents [5]. 

 Except for sarcomas possessing the tumor-specific chromosomal translocations that form the specific gene fusions such as Ewing sarcoma with EWS-FLI1 fusion gene, the genetic loci involved in sarcomagenesis have not yet been fully elucidated, even though numerous genetic changes have been reported in many kinds of sarcomas including osteosarcoma and MFH. 

 Mesenchymal stem cells (MSCs) are considered to be a potential cell-of-origin for most sarcomas, and several studies have been conducted suggesting sarcomas of MSC origin [6–8]. The genome-wide gene expression profiling in sarcomas compared to the MSCs will provide a deeper understanding of the aberrant biological pathways contributing to sarcomagenesis which may lead to the identification of diagnostic and therapeutic targets. Here we report on a genome-wide expression profiling study on rat osteosarcoma and MFH cell lines to identify genes that exhibit significant differential expression compared to MSCs isolated from syngeneic rats. Since these cells share syngeneic biological and genetic backgrounds, the differentially expressed genes and pathways identified here will possibly be culpable molecular events responsible for sarcomagenesis.

## 2. Materials and Methods

### 2.1. Cell Lines

Both rat osteosarcoma cell line COS1NR and malignant fibrous histiocytoma MFH1NR were established from chemically induced osteosarcoma in Fischer 344 rats by 4-hydroxy quinolone 1-oxide in our laboratory and revealed those bearing p53 mutation [9, 10]. Rat mesenchymal stem cells (MSCs) were isolated from rat femur bone marrow through a monolayer culture on a plastic dish [11]. COS1NR and MFH1NR cells were maintained in Dulbecco's minimum essential medium with 10% fetal bovine serum, and MSCs were maintained in Dulbecco's minimum essential medium with 15% fetal bovine serum in an atmosphere of 5% CO_2_ at 37°C.

### 2.2. Isolation and Labeling of RNA from COS1NR, MFH1NR, and MSCs

Total RNA was isolated from both COS1NR and MFH1NR cells and MSCs, using an RNeasy mini kit (Qiagen, Germany) and quality assessed by Agilent 2100 Bioanalyzer. 500 ng of total RNA was processed by Agilent expression array analysis using a Quick Amp Labeling Kit and Gene Expression Hybridization Kit. Briefly, reverse transcription was performed with primers with oligo-dT in T7 promoter to synthesize the double-stranded cDNA, then *in vitro* transcription was performed using T7 RNA polymerase to obtain Cy3-labeld cRNA.

### 2.3. Microarray Hybridization and Data Analysis

To investigate the difference in gene expression between sarcoma cells and MSCs, gene expression profiling was performed by Agilent array analysis (Agilent Technologies, Böblingen, Germany). Briefly, the Cy3-labeled cRNA were fragmented with hybridization solution and hybridized with Agilent Expression Array (Whole rat genome array) for 17 hrs at 65°C and 10 rpm and subsequently washed at room temperature. The microarray slides were scanned on an Agilent DNA Microarray Scanner. Data analysis was carried out using Agilent Feature Extraction software, analyzing pathways that were differentially expressed in sarcoma cells compared to MSCs.

## 3. Results

### 3.1. Gene Expression Profiling Distinguishes Rat Sarcomas from MSCs

In this study, oligonucleotide microarray containing probe sets for 26,930 rat genes were used to obtain genome-wide expression profiles of chemically induced rat osteosarcoma, MFH cells, and MSCs. To gain insight into the biological processes participating in sarcomagenesis, we performed pathway analysis and hierarchical clustering analysis.

**Figure 1:**
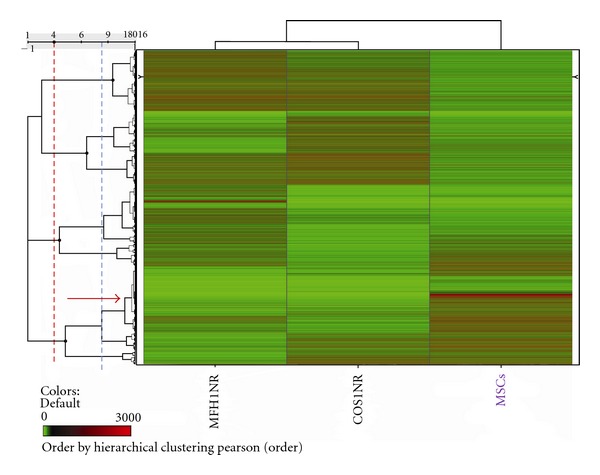
The hierarchical clustering analysis of expression profiling data clearly shows separate clusters among osteosarcoma, MFH, and MSCs.

 The hierarchical clustering analysis of expression profiling data clearly showed separate clusters among osteosarcoma, MFH, and MSCs (Figure 1). We subsequently focused on the genes strongly expressed in MSCs compared to both sarcomas for pathway analysis.

### 3.2. Differential Gene Expression Profiles Between Rat Osteosarcoma and MFH, Indicating the Altered Pathways

**Figure 2:**
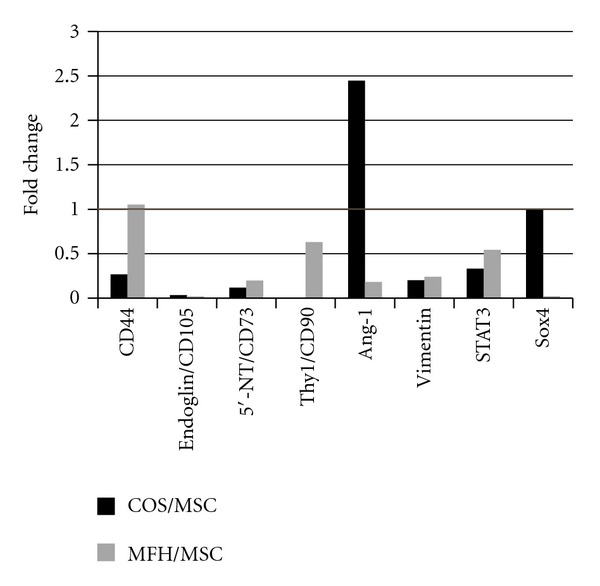
The ratio of *g* scale signal intensity of mesenchymal stem cell markers and other stem cell markers. The graph indicates the fold changes in rat sarcomas in comparison to rat MSCs.

Firstly, we analyzed stem cell markers including mesenchymal stem cell markers. Mesenchymal stem cell markers, CD44, CD73, CD90, and CD105 were all strongly expressed in MSCs compared to both sarcomas. Other stem cell markers such as STAT3 and Sox4 were also strongly expressed in MSCs, while angiopoietin-1 was highly expressed in osteosarcoma cells (Figure 2).

Genes that were differentially expressed were analyzed in the context of the pathways in which they function using the KEGG pathway map. Pathways with a higher incidence of strongly expressed genes in MSCs were cell adhesion molecules, cytokine-cytokine-receptor interaction, ECM-receptor interaction, chemokine signaling, Jak-STAT signaling, and Wnt signaling pathways. Those showing higher incidence in both sarcomas were DNA replication, cell cycle, mismatch repair, and Hedgehog signaling pathways (Table 1). 

**Figure 3:**
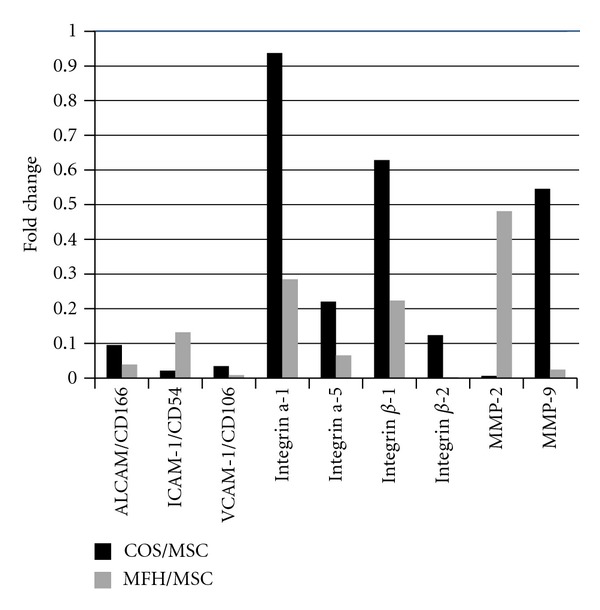
The ratio of *g* scale signal intensity of cell adhesion and extracellular matrix interaction molecules in both sarcomas compared to MSCs are shown. Integrins and their ligands CAMS were strongly expressed in MSCs, especially differential expression of CAMs between sarcomas and MSCs was significant. Matrix metalloproteinase 2 and 9 also showed higher expression in MSCs.

The ratio of *g* scale signal intensity of cell adhesion and extracellular matrix interaction molecules in both sarcomas compared to MSCs are shown in Figure 3.

Integrins and their ligands CAMS were strongly expressed in MSCs. In particular, differential expression of CAMs between sarcomas and MSCs was significant (Table 2). 

**Figure 4:**
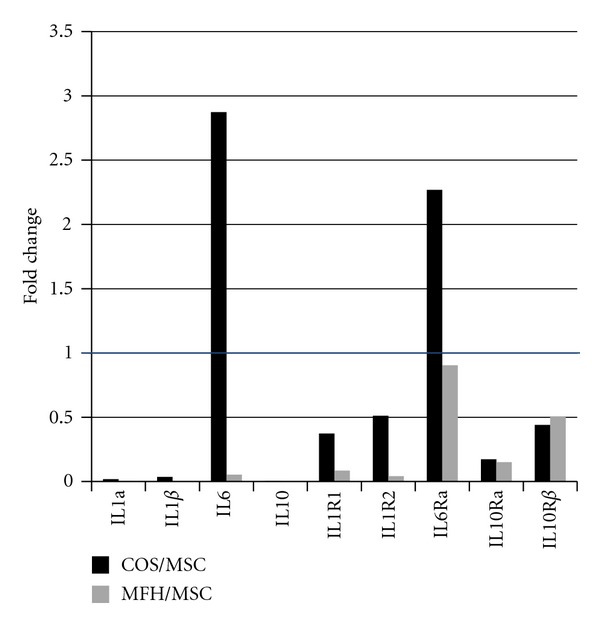
Interleukins and their receptors showed higher expression in MSCs compared to both sarcomas, except for IL6 and its receptor IL6Ra in osteosarcoma.

Matrix metalloproteinase 2 and 9 also showed higher expression in MSCs. Interleukins and their receptors also showed higher expression in MSCs compared to both sarcomas, except for IL6 and its receptor IL6Ra in osteosarcoma, suggesting the possible involvement of IL6 signaling in osteosarcoma development (Figure 4).

**Figure 5:**
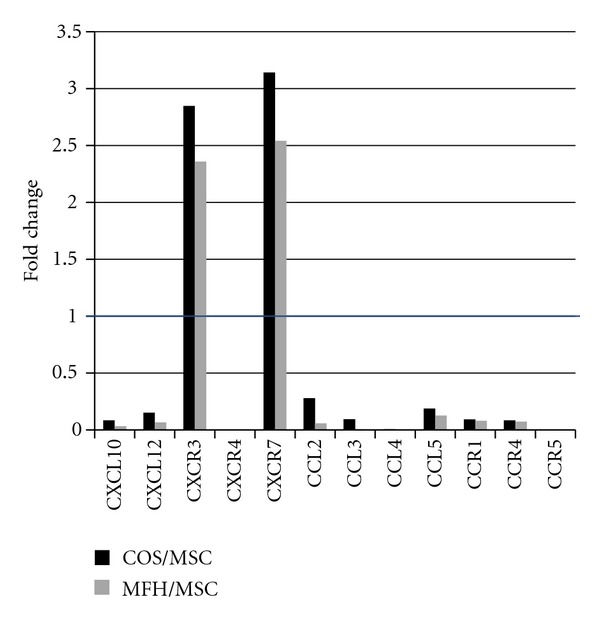
The ratio of most chemokines and their receptors also showed predominant expression in MSCs compared to both sarcomas, except for CXCR 3 and 7.

The ratio of most chemokines and their receptors also showed predominant expression in MSCs compared to both sarcomas, except for CXCR 3 and 7 (Figure 5). CXCR7 is one of the receptors for CXCL12 other than CXCR4, therefore the CXCL12-CXCR7 axis might be involved in sarcoma development through tumor cell and stromal cell interaction.

**Figure 6:**
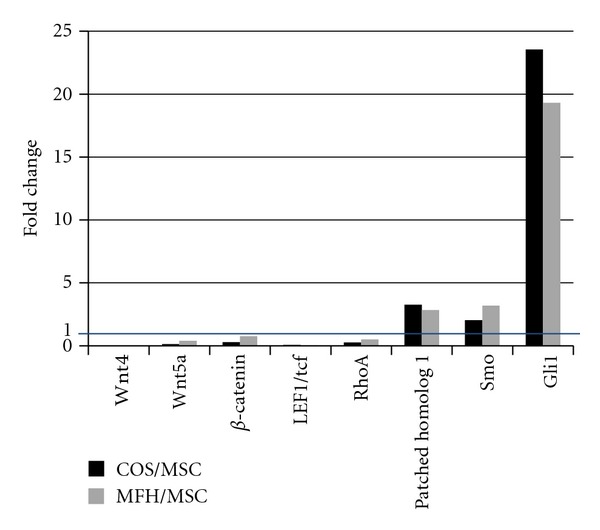
The expression of genes involved in Wnt signaling and those in Hedgehog signaling suggests the downregulation of Wnt signaling and upregulation of Hedgehog signaling pathways might be involved in rat sarcomagenesis.

Genes including Wnt 4 and 5a, beta-catenin, LEF1, and RhoA in Wnt-signaling pathways showed significantly lower expression and DKK1, a Wnt signal inhibitor showed strong expression in both sarcomas, while those in Hedgehog pathways showed higher expression in both sarcomas, especially Gli1 (Figure 6, Table 2). These data suggest that downregulation of Wnt signaling and upregulation of Hedgehog signaling pathways might be involved in rat sarcomagenesis.

**Figure 7:**
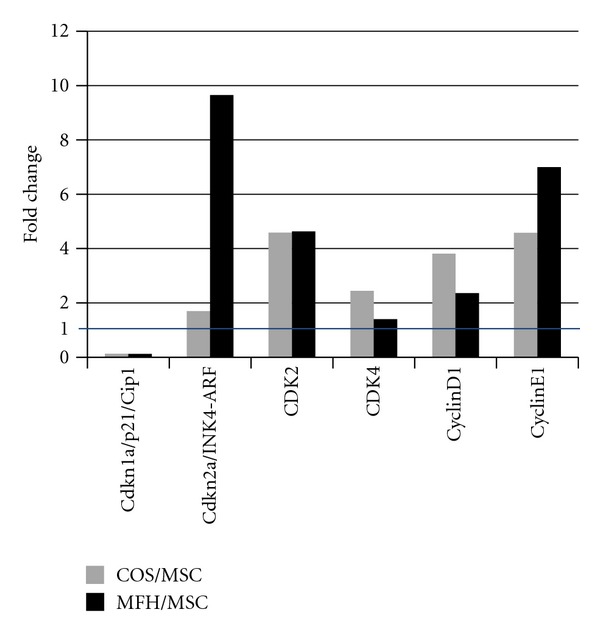
CDK inhibitor p21 showed significantly lower expression and cell cycle accelerators including CDK2 and 4, cyclinD1 and E1 were highly expressed in both sarcomas compared to MSCs.

Although cyclin-dependent kinase inhibitor p16 was highly expressed in both sarcomas, another CDK inhibitor p21 showed significantly lower expression and cell cycle accelerators including CDK2 and 4, cyclinD1 and E1 were highly expressed in both sarcomas (Table 2). This indicated that the cell cycle regulation was upregulated in sarcomas which possessed the p53 mutation [10], compared to MSCs (Figure 7).

## 4. Discussion

To identify the possible biological characteristics of osteosarcoma and MFH, a comparison of gene expression profiles with their presumed progenitors, bone marrow-derived MSCs, resulted in a large set of differentially expressed genes.

 Frequently identified genetic alterations in osteosarcoma and MFH are p53 and Rb mutation, MDM2 amplification, loss of function of p16, and CDK4-cyclinD amplification. Most of these are related to cell cycle regulation. The direct evidence for induction of sarcomas by application of these genetic changes was demonstrated by the *Prx1-Cre;p53fl/fl *mice that developed osteosarcoma, rhabdomyosarcomas as well as undifferentiated sarcomas *in vivo*. Furthermore, deletion of one Rb allele in these mice increased the frequency of osteosarcomas [12]. However, the detailed mechanisms of sarcomagenesis are yet to be fully identified. 

There have been several reports indicating that MSCs could be a potential cell of origin of several sarcomas. Hogendoorn's group reported that the spontaneous malignant transformation of MSCs producing osteosarcoma was linked to loss of p16 function [13, 14]. Others have shown that the transformation of MSC can be achieved in a more controlled situation. For instance, Riggi reported induction of Ewing sarcoma from MSCs with EWS-FLI1 introduction [15]. Rubio reported that deleting p53 in MSC induced leiomyosarcoma [16]. MFH also could be induced by inactivation of the Wnt signaling pathway or chemical treatment of 3-methylcholanthrene [17, 18]. 

Wnt signaling may have a very different role in sarcomagenesis as compared to the development of other malignancies such as colorectal carcinoma [19]. The Wnt pathway is required for osteoblast lineage determination at an early stage [20] and coordinates with Hedgehog signaling for bone formation by controlling osteoblast differentiation [21]. Furthermore, Wnt signals are transduced in a context-dependent manner to the canonical pathway for cell fate determination and to the noncanonical pathway for the regulation of planar cell polarity, cell adhesion, and motility [22, 23]. Several studies have suggested that inactivation of Wnt signaling might be involved in osteosarcoma tumorigenesis [24, 25] as well as MFH as described above. However, the involvement of the Wnt pathway in sarcomagenesis is still controversial. A recent report suggested that frequent canonical Wnt activation was observed in multiple sarcoma subtypes, and downregulation of the activated Wnt pathway inhibited sarcoma cell proliferation [26, 27]. These findings suggest that genetic alteration is independent of Wnt signaling such as p53, Hedgehog may mask the function of the Wnt pathway in sarcomagenesis, or alteration of Wnt signaling may occur at some late stage of neoplastic progression. Further studies will be required to clarify this matter.

Meanwhile, aberrant activation of Hedgehog signaling is associated with various types of cancers, in which deregulation of cellular growth, survival, and adult stem cell maintenance is believed to be involved. The Hedgehog signaling pathway initiates a signaling cascade, ultimately leading to the activation of the Gli transcription factors that mediate signal transduction to the nucleus. In contrast to the Wnt pathway, Hedgehog signaling has been reported for its activation in sarcomas through the amplification of Gli, a transcriptional factor located downstream of Hedgehog signaling [28, 29]. Hedgehog signaling is also involved in cell cycle regulation. Overexpression of Gli2 in human MSCs increases cell proliferation and progression through cell cycle regulation, and inhibition of Gli2 disrupted the growth of osteosarcoma cells [30]. Several reports have suggested a critical role for p53 on the tumorigenic effects of Hedgehog pathway activation [31, 32]. Although our data could not find overexpression of MDM2 in sarcomas, Gli1 has been reported to repress p53 through the activation of MDM2 [33]. In addition, the disruption of Hedgehog signaling by small interfering RNA against Gli1 induced p21/Cip1 expression that resulted in suppression of cell proliferation in a p53-independent mechanism [34]. Our data support the findings that the Wnt and Hedgehog signaling pathways in human sarcomas could be adapted in the rat sarcoma model as well. 

Several reports have claimed that surface markers for mesenchymal stem cells such as CD44 and CD90 were expressed in human osteosarcomas [35–37]. Yet, our data indicated that the expression of those surface markers for mesenchymal stem cells was lower in sarcoma cells compared to MSCs. However, the levels of CD44 and CD90 expression in MFH were very similar to MSCs compared to osteosarcoma. Therefore, a possible explanation for this discrepancy could be the down-regulation of the expression of stem cell markers after differentiation into mature mesenchyme cells such as osteoblasts [38]. Another explanation is that the strong expression of stem cell markers could be detected in a subset of the population, so-called “cancer stem cells”, so that the level of expression in these cell populations might be compatible to that in MSCs. Further study will be required on this matter.

**Figure 8:**
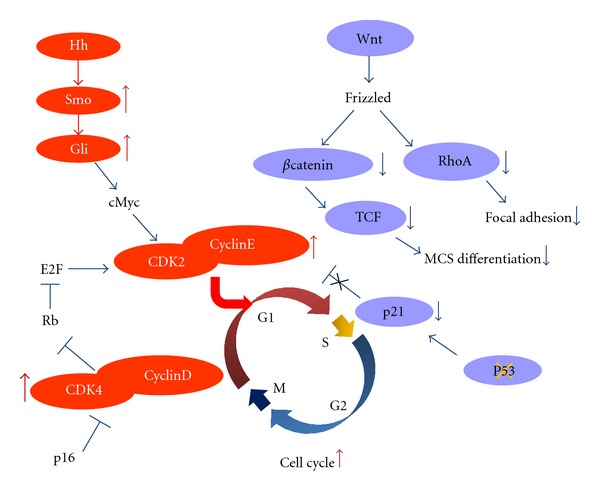
A summary of pathways with differentially expressed genes comparing sarcomas to MSC in the current study. Disruption of p53 and activation of Hedgehog signaling may lead to cell cycle acceleration through downregulation of p21 and upregulation of CDK and cyclins. The inactivation of the Wnt signaling pathway may lead to the deregulation of differentiation as well as focal adhesion function in MSC, which might be required for normal stem cell maintenance.

Pathways with differentially expressed genes comparing sarcomas to MSC in the current study are summarized as shown in Figure 8. Disruption of p53 such as point mutation or MDM2 amplification and activation of Hedgehog signaling may lead to cell cycle acceleration through downregulation of p21 and upregulation of CDK and cyclins. In addition, inactivation of the Wnt signaling pathway may lead to the deregulation of differentiation as well as focal adhesion function in MSC, which might be required for normal stem cell maintenance.

Taken together, these findings have led us to the hypothesis of a model for sarcomagenesis. Wnt and Hedgehog signaling may change during the process of differentiation and might result in cell cycle acceleration in conjunction with p53 mutations as well as DNA replication. An advantage for this study would be that the cells originated from syngeneic rats, indicating that they share the same genetic background. The limitations of this study would be the use of cultured cells at first, which might cause genetic alterations, then the potential presence of cellular heterogeneity, especially in MSCs that were not sorted with specific cellular surface markers. Secondly, further study will be required to validate and confirm these microarray data with different methods such as RT-PCR. Finally, the functional analysis of differentially expressed genes, especially Hedgehog and Wnt signaling, will definitely be required in any future study. 

## 5. Conclusions

 We have found that stem cell markers as well as Wnt signaling pathways were downregulated in sarcomas, while Hedgehog pathways and cell cycle regulation were upregulated. Other pathways might possibly be involved in sarcomagenesis, thus further study will be required including confirmation and functional analysis for differentially expressed genes.

## Figures and Tables

**Table 1:** Pathways with higher incidence of strongly expressed genes.

Rat MSCs ↑	*P *value
Cell adhesion	<0.0001
ECM-receptor interaction	<0.0001
Cytokine-cytokine-receptor interaction	<0.0001
Chemokine signaling	<0.0001
Jak-STAT signaling	= 0.0004
Wnt signaling	= 0.0224

Rat Sarcomas ↑	*P *value

DNA replication	<0.0001
Cell cycle	= 0.0005
Mismatch repair	= 0.0020
Hedgehog signaling	= 0.0064

**Table 2:** Genes expressed significantly differentiate between rat sarcomas and MSCs.

Gene	COS/MSC	MFH/MSC
ALCAM/CD166	0.09	0.04
ICAM-1/CD54	0.02	0.13
VCAM-1/CD106	0.03	0.01
Wnt4	0.01	0.01
LEF1/tcf	0.07	0.03
DKK1	1.27	18.65
Gli1	23.54	19.30
Cdkn1a/p21/Cip1	0.12	0.12
CDK2	4.55	4.63
CDK4	2.44	1.40
cyclinD1	3.81	2.35
cyclinE1	4.58	7.00

The values indicate the fold changes of *g* Scale signal intensities in rat sarcomas comparing to MSCs.
